# Evaluation of skin dose associated with different frequencies of bolus applications in post-mastectomy three-dimensional conformal radiotherapy

**DOI:** 10.1186/1756-9966-28-41

**Published:** 2009-03-24

**Authors:** Fundagul Andic, Yasemin Ors, Rima Davutoglu, Sule Baz Cifci, Emine Burcin Ispir, Mehmet Ertugrul Erturk

**Affiliations:** 1 Department of Radiation Oncology, Faculty of Medicine, Gaziantep University, Gaziantep, Turkey; 2 Department of Radiation Oncology, Faculty of Medicine, Hacettepe University, Ankara, Turkey

## Abstract

**Background:**

The study aimed to calculate chest-wall skin dose associated with different frequencies of bolus applications in post-mastectomy three-dimensional conformal radiotherapy (3D-CRT) and to provide detailed information in the selection of an appropriate bolus regimen in this clinical setting.

**Methods:**

CT-Simulation scans of 22 post-mastectomy patients were used. Chest wall for clinical target volume (CTV) and a volume including 2-mm surface thickness of the chest wall for skin structures were delineated. Precise PLAN 2.11 treatment planning system (TPS) was used for 3D-CRT planning. 50 Gy in 25 fractions were prescribed using tangential fields and 6-MV photons. Six different frequencies of bolus applications (0, 5, 10, 15, 20, and 25) were administered. Cumulative dose-volume histograms were generated for each bolus regimen. The minimum, maximum and mean skin doses associated with the bolus regimens were compared. To test the accuracy of TPS dose calculations, experimental measurements were performed using EBT gafchromic films.

**Results:**

The mean, minimum and maximum skin doses were significantly increased with increasing days of bolus applications (p < 0.001). The minimum skin doses for 0, 5, 10, 15, 20, and 25 days of bolus applications were 73.0% ± 2.0%, 78.2% ± 2.0%, 83.3% ± 1.7%, 88.3% ± 1.6%, 92.2% ± 1.7%, and 93.8% ± 1.8%, respectively. The minimum skin dose increments between 20 and 25 (1.6% ± 1.0%), and 15 and 20 (4.0% ± 1.0%) days of bolus applications were significantly lower than the dose increments between 0 and 5 (5.2% ± 0.6%), 5 and 10 (5.1% ± 0.8%), and 10 and 15 (4.9% ± 0.8%) days of bolus applications (p < 0.001). The maximum skin doses for 0, 5, 10, 15, 20, and 25 days of bolus applications were 110.1% ± 1.1%, 110.3% ± 1.1%, 110.5% ± 1.2%, 110.8% ± 1.3%, 111.2% ± 1.5%, and 112.2% ± 1.7%, respectively. The maximum skin dose increments between 20 and 25 (1.0% ± 0.6%), and 15 and 20 (0.4% ± 0.3%) days of bolus applications were significantly higher than the dose increments between 0 and 5 (0.2% ± 0.2%), 5 and 10 (0.2% ± 0.2%), and 10 and 15 (0.2% ± 0.2%) days of bolus applications (p ≤ 0.003). The TPS overestimated the near-surface dose 10.8% at 2-mm below the skin surface.

**Conclusion:**

In post-mastectomy 3D-CRT, using a 1-cm thick bolus in up to 15 of the total 25 fractions increased minimum skin doses with a tolerable increase in maximum doses.

## Background

Post-mastectomy radiotherapy improves survival and local control in patients with high risk breast cancer [1,2]. The chest wall is the most frequent site of recurrence and delivering adequate radiation doses to the chest wall is crucial to reducing the risk of treatment failure [3]. Keeping radiation-induced side effects as low as possible, while providing the intended dose to the chest wall remains a challenge [4,5]. Definition of surface and superficial chest wall doses provides valuable information for avoiding near-surface recurrences and limiting severe early and late skin reactions.

Tissue-equivalent material boluses, which are thick enough to provide an adequate dose build-up in the skin and superficial chest wall, are commonly used during post-mastectomy radiotherapy. Skin dose contributions of boluses and the dose delivered to skin and subcutaneous tissue are important, especially in locally advanced breast cancer [6]. The American Society of Clinical Oncology published treatment guidelines for post-mastectomy radiotherapy in 2001. These guidelines stated that the chest wall should be treated adequately but they did not comment on the use of boluses [7].

To our knowledge, the mean, minimum, and maximum skin doses associated with different durations of bolus applications have not been reported. The purpose of this prospective dosimetric study was to calculate the chest-wall skin dose associated with various frequencies of bolus applications in post-mastectomy three-dimensional conformal radiotherapy (3D-CRT) and to provide detailed information to aid in the selection of an appropriate bolus regimen in this clinical setting.

## Methods

### CT simulation

We performed CT-simulation of 22 patients immobilized with a breast-board. Each patient was positioned supine on the breast board with the ipsilateral arm abducted above the head; board angles were tailored according to the patient's anatomy. Patients were scanned with a 6 detector helical CT (CT Brilliance, Philips Medical Systems, Netherlands) with 5-mm slices from mid-neck to mid-abdomen.

### Volumes of interest

**Figure 1:**
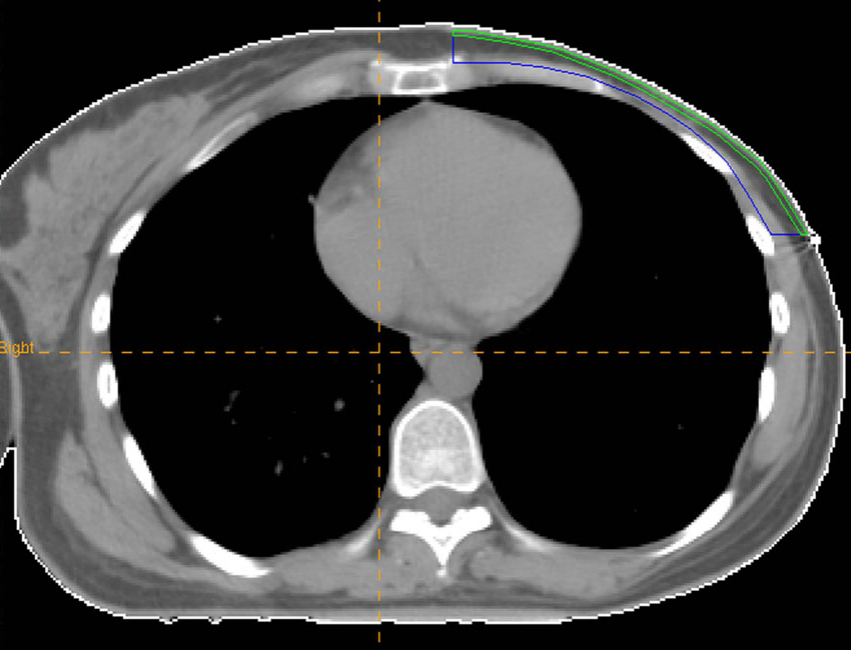
**Skin structure (green line) and clinical target volume (dark-blue line)**.

The external surface of the patient and lung contours were defined by automated density gradient tracking then edited and verified by physicians FA and RD. The chest wall for the clinical target volume (CTV) was delineated on corresponding transverse CT images (Figure 1) by FA and RD using the external skin surface anteriorly, the rib-soft tissue interface posteriorly, the inferior aspect of the clavicular head superiorly and 1-cm below the contralateral inframammary fold inferiorly. Medial and lateral borders of the CTV were delineated considering lateral border of the sternum and the mid-axillary line, respectively.

To evaluate skin dose accurately, another volume including 2-mm surface thickness of the CTV was contoured (Figure 1) as skin structure.

The planning target volume (PTV) was defined by adding 5-mm to the CTV. However, the superficial contour of the PTV was outlined 3-mm deep to the skin surface since the build-up effect would cause apparent underdosage in the dose-volume histograms (DVH) and difficulties in the evaluation of the treatment plans.

### 3D-CRT planning

The Precise PLAN^®^2.11 (Elekta, Crawley, UK) treatment planning system (TPS) was used for 3D-CRT planning. The TPS calculates the dose distribution of the photon beam using an irregular field algorithm based on data measures in a phantom for different depths and field sizes. The irregular field algorithm takes into account the tissue inhomogeneity and uses an integration scheme to evaluate the scatter component of the dose.

**Figure 2:**
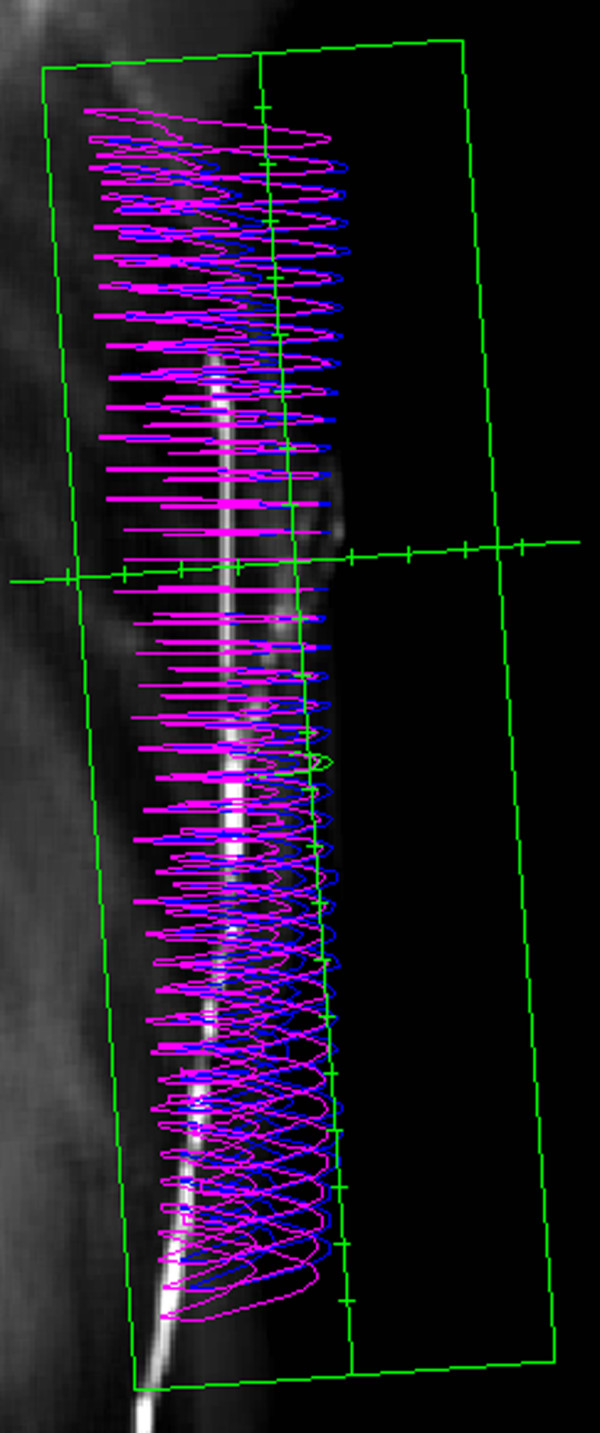
**Tangential radiation field on digital reconstructed radiograph**.

Two opposed tangential radiotherapy fields were created (Figure 2). The beam centre was located in the chest wall. To reduce the irradiated lung volume, incident beam angles were used to match the fields at the dorsal field edge non-divergently and lung tissue was shielded when necessary. The nominal prescribed dose was 50 Gy in 25 fractions using 6-MV photons. The calculated dose was normalized to a relevant point in the PTV to provide dose homogeneity.

Although a uniform dose to the CTV within 95% to 107% of the prescribed dose is recommended, a variation of plus or minus 10% from the prescribed dose is widely used in clinical practice [8]. In the present study, to accurately evaluate the dose contribution of later bolus applications, we planned that 90% to 110% of the prescribed dose to the PTV would be delivered before the bolus applications. Maximum doses higher than 110% of the prescribed doses were ignored if they encompassed a point and not a volume.

**Figure 3:**
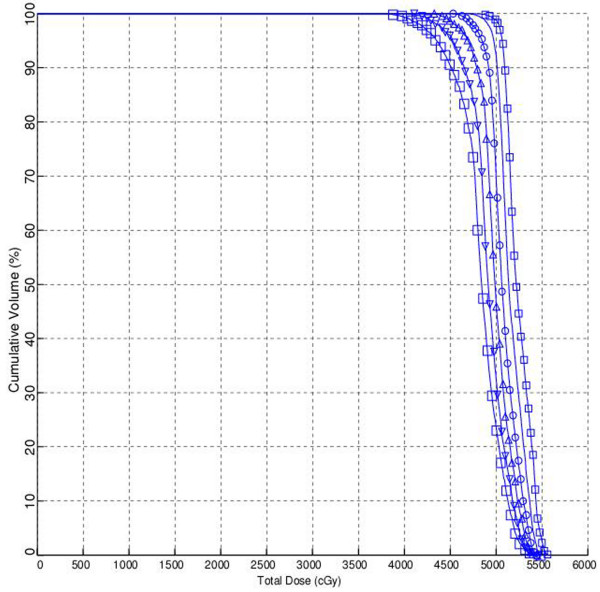
**The dose-volume histograms of skin structures according to days of bolus applications in one case**. (White square) – 0 days; (upside down white triangle) – 5 days; (white triangle) – 10 days; (White circle) – 15 days; (horizontal line) – 20 days; (small white square) – 25 days of bolus applications.

A 1-cm thick bolus with a 1 gr/cc density was placed over the chest wall for 0, 5, 10, 15, 20, or 25 treatment days in TPS calculations for all patients. Cumulative DVHs were generated for each bolus regimen and for each patient. The size of the dose bin used for the DVH calculation was 0.01 Gy. The DVHs of skin structures for 0, 5, 10, 15, 20 and 25 days of bolus applications in one case are shown in Figure 3.

### Dosimetric Analysis

To test the accuracy of TPS near-surface dose calculations, solid plate phantom (Iba Dosimetry, Schwarzenbruck, Germany) and EBT gafchromic (International Specialty Products, Wayne, NJ, USA) films were used for both calibration and experimental measurements at a Synergy Platform 6-MV linear accelerator (Elekta, Crawley, UK).

For calibration, 4 × 4 cm^2 ^films were irradiated at 100-cm fixed SSD (source-to-skin distance) and 5-cm depth with different doses ranging from 4.128 cGy (5 MU) to 336.1 cGy (400 MU). After 24 hours later, irradiated films were scanned using Epson, Expression 10000 XL (Seiko Epson Corporation, Japan) scanner, read with Mephysto mc^2 ^v1.3 (PTW, Freiburg, Germany) software and optic density-dose calibration curves were obtained.

For dose measurements, 4 × 4 cm^2 ^films were placed at the centre of the 10 × 10 cm^2 ^field at specific depths (0, 1, 2, 3, 4, 5, 6, 8, 10, 12, 15, 20, 25 and 30-mm) and irradiated at 100-cm fixed SSD with a dose of 83.25 cGy (100 MU).

### Statistical analysis

We analyzed the dosimetric data of skin structure for all treatment regimens. The mean, minimum and maximum doses to skin in all bolus regimens were compared by the Friedman test and Wilcoxon analysis using Statistical Package for Social Sciences (SPSS), version 16.0. P-values of 0.05 or less were considered statistically significant. Values are expressed as mean (range) ± standard deviation (SD) and percent of prescribed dose.

## Results

The mean, minimum and maximum PTV doses before the bolus applications were 101.8% (100.2–103.2%) ± 0.9%, 91.2% (90.0–94.5%) ± 1.2% and 109.4% (105.0–110.6%) ± 1.3%, respectively.

**Figure 4:**
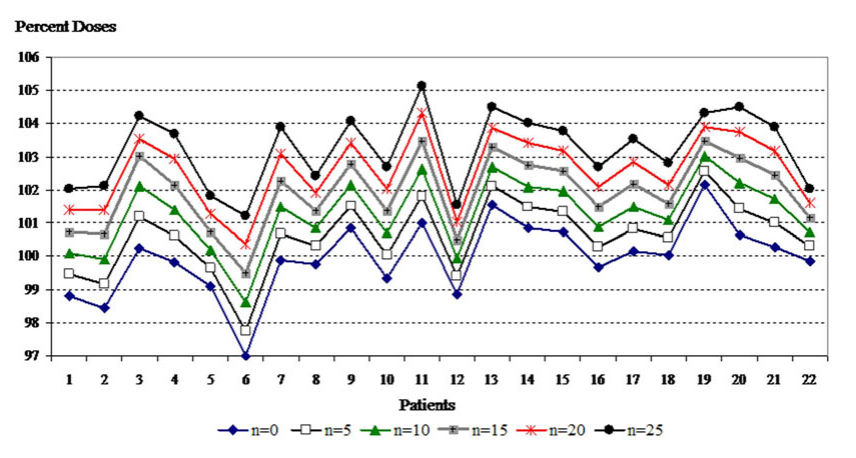
**Mean values of skin structure doses according to bolus frequencies for all plans**.

**Figure 5:**
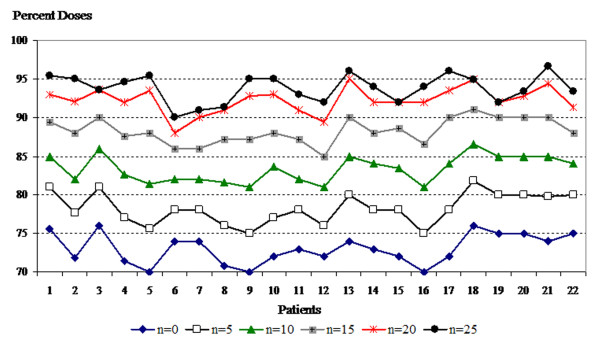
**Minimum values of skin structure doses according to bolus frequencies for all plans**.

**Figure 6:**
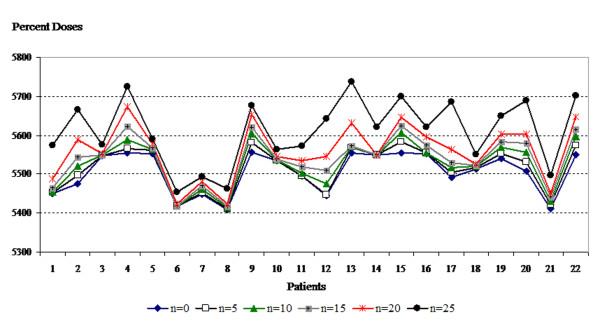
**Maximum values of skin structure doses according to bolus frequencies for all plans**.

Table 1 shows the mean, minimum, and maximum doses to the skin according to days of bolus application. These doses were significantly (p < 0.001) increased with increased days of bolus application. The mean, minimum and maximum doses to the skin structure with each bolus regimen and in each plan are shown in Figures 4, 5 and 6.

**Table 1:** Mean values of mean, minimum, and maximum skin structure doses according to bolus frequencies

**Bolus Regimen**	Mean ± SD*	Minimum ± SD*	Maximum ± SD*
0	100.0 ± 1.1	73.0 ± 2.0	110.1 ± 1.1
5	100.6 ± 1.1	78.2 ± 2.0	110.3 ± 1.1
10	101.3 ± 1.1	83.3 ± 1.7	110.5 ± 1.2
15	101.9 ± 1.1	88.3 ± 1.6	110.8 ± 1.3
20	102.6 ± 1.1	92.2 ± 1.7	111.2 ± 1.5
25	103.2 ± 1.1	93.8 ± 1.8	112.2 ± 1.7

* as percent of prescribed dose; SD, standard deviation

Bolus use in all fractions provided a 20.8% ± 2.8% minimum skin dose increment. The minimum skin dose increments between 20 and 25 (1.6% ± 1.0%), and 15 and 20 (4.0% ± 1.0%) days of bolus applications were significantly lower than the dose increments between 0 and 5 (5.2% ± 0.6%), 5 and 10 (5.1% ± 0.8%), and 10 and 15 (4.9% ± 0.8%) days of bolus applications (p < 0.001). Furthermore, the minimum skin dose increment between 20 and 25 (1.6% ± 1.0%) days of bolus application was lower than the dose increment between 15 and 20 (4.0% ± 1.0%) days of bolus application (p < 0.001).

Bolus use in all fractions resulted in a 2.0% ± 1.2% maximum skin dose increment. The maximum skin dose increments between 20 and 25 (1.0% ± 0.6%), and 15 and 20 (0.4% ± 0.3%) days of bolus applications were significantly higher than the dose increments between 0 and 5 (0.2% ± 0.2%), 5 and 10 (0.2% ± 0.2%), and 10 and 15 (0.2% ± 0.2%) days of bolus applications (p ≤ 0.003). Furthermore, the maximum skin dose increment between 20 and 25 (1.0% ± 0.6%) days of bolus application was higher than the dose increment between 15 and 20 (0.4% ± 0.3%) days of bolus application (p < 0.001).

The dose increase of the mean values between all bolus frequencies was similar (p= 0.965).

Measurements using EBT gafchromic film revealed that Precise PLAN^®^2.11 TPS overestimated near-surface dose 10.8% at 2-mm below the skin surface.

## Discussion

Bolus thickness required to enhance surface dose is optimized according to surface and build-up region dosimetry. In the present study, a 1-cm bolus was used to increase skin doses. This thickness was chosen because 6-MV photon energy with a 1.5-cm maximal depth was used for tangential fields.

The skin dose contributions of 1-cm bolus material during whole or a part of treatment duration were calculated in this study. The results showed a trend of increasing minimum skin dose when the days of bolus application were increased. The minimum skin dose increments were expected to be linear among the bolus durations. However, the minimum skin dose increments between 20 and 25 (1.6% ± 1.0%), and 15 and 20 (4.0% ± 1.0%) days of bolus applications were significantly lower than the dose increments between 0 and 5 (5.2% ± 0.6%), 5 and 10 (5.1% ± 0.8%), and 10 and 15 (4.9% ± 0.8%) days of bolus applications while the maximum skin dose increments were significantly higher. TPS dose calculation algorithm and treatment related factors such as delivery technique, field size and angle of beam incidence are supposed to be associated with these non-linear dose increments. Therefore, our results need to be clarified in further dosimetric studies using different TPS, techniques, beam energies, and bolus thicknesses.

Determining the necessary frequency of bolus treatments is critically important in post-mastectomy radiotherapy, since it influences the irradiated volume as well as the skin doses. Although the literature contains several recommendations for radiotherapy planning techniques, there are few recommendations regarding bolus use [4,5,9-11]. The optimal duration and the optimal thickness of the bolus material still remain uncertain and change centre to centre [7,12]. Wide regional variations in the use of boluses were reported by Vu et al. in an international survey of radiation oncologists and their opinions on the indications for boluses in post-mastectomy radiotherapy [12].

Determining the difference between the calculated and measured surface dose is useful when evaluating and comparing patient plans and also when optimizing the use of boluses. Many factors affect the magnitude of the surface dose, such as the delivery technique, field size, angle of beam incidence, air gap and the use of bolus material and beam modifiers [13-15]. Calculation of skin doses is difficult in most TPSs due to their inability to account for all the factors that contribute to the surface dose. However, the Monte Carlo TPSs and, to a lesser extent, the modern true 3D algorithms are able to calculate skin doses [16-18]. Doses calculated with different TPSs have been reported to underestimate and overestimate measured skin doses [15,19-23]. Measured skin doses also may differ according to the dosimetry used [13]. In the present study, EBT gafchromic films were used to test the accuracy of TPS and experimental measurements were performed at 10 × 10 cm^2 ^field at 100-cm fixed SSD since same tissue equivalent field as in a post-mastectomy patient could not be created to measure depth doses. The measurements revealed that Precise PLAN^®^2.11 TPS overestimated the near surface dose comparable to the literature [15,19-22]. However, the goal of the present study was to reveal the trend in proportional skin doses with various frequencies of bolus applications, using the same TPS to calculate doses to the same skin structures.

The thickness of the epidermis varies between 0.05–1.5 mm, depending on the anatomic location. The International Commission on Radiological Protection and the International Commission on Radiation Units and Measurements recommends a depth of 0.07-mm, corresponding to the epidermal and dermal layers, for practical skin dose assessments [24,25]. Measuring the dose at that depth is very difficult. Therefore, in the present study, skin structure was defined as 2-mm surface thickness of the CTV. Court et al. also used a 2-mm thick skin structure in their investigation of the accuracy of skin dose calculations on a semi-cylindrical model of a neck or breast [15].

The superficial PTV contour is usually outlined 5-mm deep to the skin surface to avoid apparent under-dosage in the DVH due to build-up effects [4,26]. Although this is reasonable in breast conserving surgery, it may result in wrong dose-volume information in post-mastectomy radiotherapy, particularly in locally advanced breast cancer when the skin is close to or included in the target volume. Therefore, we believe that delineating a skin structure in addition to the CTV and PTV would provide important information in post-mastectomy treatment planning. Furthermore, surface dose measurements for the comparison of calculated and measured skin doses would also help to define accurate skin dose deficit.

## Conclusion

In post-mastectomy 3D-CRT, using a 1-cm thick bolus in 5, 10, and 15 of the total 25 fractions increased minimum skin doses with a tolerable increase in maximum doses. Hence, up to 15 days of bolus applications appear to be the optimal bolus regimens. However, while deciding duration of bolus application, the difference between calculated and measured skin doses should also be considered, besides the calculated skin dose deficit in the TPS.

## Abbreviations

3D-CRT: three-dimensional conformal radiotherapy; CTV: clinical target volume; TPS: treatment planning system; PTV: planning target volume; DVH: dose-volume histogram; SPSS: Statistical Package for Social Sciences; SD: standard deviation

## Competing interests

The authors declare that they have no competing interests.

## Authors' contributions

FA conceived of the study, coordinated the study, edited and verified the external surface of the patient and lung contours, delineated target volumes, helped acquisition of data, performed the statistical analysis and draft the manuscript. YO has performed treatment plans, participated in acquisition of data and helped to draft the manuscript. RD edited and verified the external surface of the patient and lung contours, delineated target volumes, participated in acquisition of data and helped to draft the manuscript. SB has performed treatment plans, participated in acquisition of data and helped to draft the manuscript. EBI has performed treatment plans and experimental measurements, helped acquisition of data and drafting the manuscript. MEE involved in experimental measurements and data analysis and helped to draft the manuscript. All the authors read and approved the final manuscript.
